# Standardization of Rn-222 at the Australian Radiation Laboratory

**DOI:** 10.6028/jres.095.020

**Published:** 1990

**Authors:** T. H. Gan, S. B. Solomon, J. R. Peggie

**Affiliations:** Australian Radiation Laboratory, Lower Plenty Road, Yallambie, Victoria, 3085, Australia

**Keywords:** Rn-222 calibration, scintillation cell, uncertainty components

## Abstract

The standardization of Rn-222 at the Australian Radiation Laboratory involves the calibration of scintillation cells by two methods using standard Ra-226 solutions traceable to the National Institute of Standards and Technology. One of these methods, namely the injection method, involves direct transfer of Rn-222 into a scintillation cell. In the other method, known as the volumetric method, the Rn-222 is flushed into a large container and the scintillation cell calibrated by sampling from this container. A comparison of the two methods showed that similar results were obtained, with the overall random uncertainty being 3.4% for one standard deviation. Using better estimates of the true calibration factors, the overall random uncertainty was reduced to 1.8% for one standard deviation.

## 1. Introduction

In 1983 the Nuclear Energy Agency (NEA) of the Organization for Economic Cooperation and Development (OECD) and the Commission of European Communities (CEC) jointly organized an international intercomparison program for measurements of radon, thoron, and daughter products [1]. The Australian Radiation Laboratory (ARL) was designated as the reference laboratory for the Pacific region. The primary method for Rn-222 measurements at ARL is based on scintillation cells which have been calibrated by Ra-226 solutions traceable to the National Institute of Standards and Technology (NIST). This report describes the calibration methodologies used in the Asian/Australasian Region Intercomparison for Radon which was completed in September 1988 [2].

## 2. Methodology

### 2.1 Injection Technique

The scintillation cells were calibrated at ARL using two complementary techniques, namely the direct or injection method and the volumetric method. In the injection method the scintillation cells were filled with a known activity of Rn-222 by bubbling radon-free air through a glass vessel containing a standard solution of Ra-226. The amount of Rn-222 present was calculated knowing the time of build-up since the solution was last flushed. The transfer of Ra-222 was effected by evacuating a scintillation cell (nominally of volume 0.14 L) and attaching it to the outlet valve on the standard solution container. Aged compressed air or nitrogen admitted from the inlet port was used to transfer the Rn-222 by bubbling through the source until the scintillation cell was at atmospheric pressure. The pressure was monitored at the outlet valve with an electronic pressure sensor (Druck^1^). Typical periods of approximately 20 min were required for completion of the transfer procedure. The scintillation cells were then counted using 30 repeated measurements of 10 min duration and a calibration factor to convert counts to concentration (Bq m^−3^) was obtained from the known activity and cell volume. All 16 ARL cells were individually calibrated in this manner. However, repeat calibrations were obtained for some cells, in which case, the mean calibration factor was used as the individual calibration factor for the particular cell, with the standard deviation providing a measure of the random uncertainty due to the variability in the calibration procedure.

### 2.2 Volumetric Method

In the volumetric method the Rn-222 is transferred into a partially evacuated large bottle of volume about 1 L. Prior to calibration, the bottle was flushed with compressed air or nitrogen. On completion of Rn-222 transfer the Rn-222 to be sampled was slighty over pressure. After sampling, the scintillation cell pressure was equalized to atmospheric pressure. This method has two distinct advantages compared to the injection method. The first is that the volume of the bottle is known more accurately than the cell volume. The second advantage is that a much larger volume of air can be bubbled through the source, thus ensuring a more complete transfer of Rn-222. A disadvantage is that an additional transfer vessel is required, increasing the possibility of Rn-222 leakage, particularly since the vessel is overpressurized. No indications of system leakage were observed.

### 2.3 Reduction of Calibration Variability

The determination of the individual calibration factors of the set of 16 ARL cells using the above methods were influenced by physical differences between cells. These differences were attributable to two factors. The first factor is nonuniformities in the phosphor coating among the cells. The component of random uncertainty due to the resultant variability in scintillation response was estimated by filling each cell with the same radon concentration from a large Rn-222 chamber and analyzing the results of each cell using a calibration factor of unity for all cells.

The second factor is systematic differences in counting response in the bank of four photomultipliers used for simultaneous counting of cells in groups of up to four cells. During counting of the calibration samples, the 16 cells were divided into four separate groups, labelled N1–N4, with the individual cells of any group being considered as having a particular photomultiplier response. The appropriate relative response for each of the four photomultiplier tubes was determined by counting the one cell containing a known sample of radon gas in each of the four photomultiplier counting assemblies. In this manner, the effect on individual calibration factors so obtained was minimized.

However, the combined effect of the two responses resulted in small systematic biases in the calibration factors. The biases were observed as Rn-222 meaurement variability which was larger than the random uncertainty expected from counting statistics only. This variability was reduced by using individual calibration factors which had been adjusted for the photomultiplier response of the cell relative to the mean response, assuming that the mean calibration factor was the best estimate of the true calibration factor. These new calibration factors, *Y*_rel_, based on the relative sensitivities of individual cells, were calculated from

$$Y_{\text{rel}}=\frac{\text{Cell}\;\text{Counts}}{\text{Mean}\;\text{Cell}\;\text{Counts}}\cdot Y_{\text{mean}}$$ (1)

and were experimentally determined by filling each scintillation cell with the same concentration of gas from a large Rn-222 chamber.

## 3. Results

### 3.1 Injection Technique

The results for one cell calibrated using this technique are shown in table 1. The numbers in the columns refer to replicate calibrations of the same cell and the mean and standard deviation (1*s_i_*) were obtained from the repeat determinations. In this case, for the same cell having a nominal volume of 0.14 L, the mean of four calibrations was determined to be 2260×10^−6^ counts s^−1^ (Bq m^−3^)^−1^ [0.699 counts min^−1^ (pCi 0.14 L^−1^)^−1^] with the variability in the calibration procedure being 2.3% for 1*s_i_*.

### 3.2 Volumetric Method

The calibration factors of a single scintillation cell obtained by using the volumetric method are also shown in table 1. The mean of three replicate determinations was 2270×10^−6^ counts s^−1^ (Bq m^−3^)^−1^ [0.701 counts min^−1^ (pCi 0.14 L^−1^)^−1^], with a random uncertainty of 2.4% for 1*s_i_*. Both the mean and uncertainty compare favorably with the values obtained from the injection technique, the difference in means being 0.3%; clearly both techniques are comparable in accuracy and precision. However, as discussed below, due to the small volumes involved in the injection method, there was incomplete transfer of Rn-222 in some cases, leading to the rejection of some erroneous results. This action was unnecessary in the volumetric method, indicating a more reliable transfer of Rn-222 into the larger volume.

Since the individual measurements of Rn-222 using scintillation cells were also influenced by physical differences between cells, this component of variability was estimated (sec. 2.3) using results from cells which were exposed to the same Rn-222 concentration and analyzed using a calibration factor of unity for all cells. These results are listed in table 2, and exclude the component of systematic differences due to individual counting response of the four photomultipliers.

### 3.3 Reduction In Calibration Variability

The measurements of Rn-222 concentration using the original calibration factors determined by the volumetric method are shown in table 3 and are to be compared with the measurements of the same Rn-222 samples using the adjusted calibration factors as shown in table 4. The numbers in the columns are the measured concentrations with each group (N1–N4) comprising four replicate measurements each. The grand means and grand *s_i_* were determined from the 16 replicate measurements. It can be seen from tables 3 and 4 that the grand means were virtually identical, the difference being 0.4%. However, the overall random uncertainty or grand *s_i_* obtained using the old calibration factors was 3.4%, which is significantly larger than the value of 1.8% using the adjusted calibration factors.

## 4. Discussion

### 4.1 Injection Technique

The sources of random uncertainty applicable to the technique include components which have been described in the previous section. Since calibrations at ARL are now standardized on the volumetric method, further discussion is confined to components of interest.

#### 4.1.1 Variability In Calibration Procedure

This was determined (table 1) from four repeat measurements on the same scintillation cell to be 2.3% for 1*s_i_*. However this value was obtained from measurements in which erroneous results were excluded.

#### 4.1.2 Variability Between Scintillation Cells

From table 2, the determination of this component from 16 replicate measurements was 2.6% for 1*s_i_*.

#### 4.1.3 Efficiency of Rn-222 Removal by Bubbling

The potential for a less reliable transfer of Rn-222 mainly associated with the injection technique arises from the small volume (0.14 L) of air passing through the Ra-226 solution, hence the possibility that some Rn-222 may not have been transferred to the cell. Measurements were carried out to determine the fraction not transferred. A second scintillation cell was used to measure the amount left behind in the solution by flushing with air immediately after a calibration transfer. A small percentage (0.87%) of Rn-222 was found in this second cell. While this amount may be a small percentage of the present transfer in absolute terms, it may constitute a substantial fraction of the next transfer. For example, if the Ra-226 standard solution is bubbled when the Rn-222 is close to full strength, the small remaining fraction will contribute up to 14% if the source is reused within 8 h. In practice, these calibration results were rejected, but the potential for small systematic biases of the order of a few percent still exists. These biases are then observed as Rn-222 concentration variability between cells and variability between repeat calibrations for any individual cell. The approach based on mean and relative sensitivity adjustment of individual calibration factors should minimize the effect of such biases.

#### 4.1.4 Estimate of Volume of Cell

This quantity applies to the injection technique only. Since the cell calibration is intended to provide the user with an estimate of the cell response per unit concentration of Rn-222 in the environment the injection method is only valid providing the volume of the scintillation cell is known accurately. The volume of the cells was measured by filling a sample cell with water and weighing the water to determine the volume of the cell. The cell volume was determined with an accuracy of 0.3% from nine repeat measurements. It was assumed this cell was representative of the batch of 16 cells used. Cell volume variability is believed to be less than 1%.

### 4.2 Volumetric Method

#### 4.2.1 Variability in Calibration Procedure

The random uncertainty of 2.4% for 1*s_i_* was in good agreement with that found for the injection technique. Since the Rn-222 transfer was more reliable using the volumetric method, other sources contributing to this variability should be investigated. The most likely contributor is the variability in residual Rn-222 in the solution and dead space, which was caused by the variability in effective volume transferred due to the partial evacuation of the bottle. Perhaps complete evacuation of the bottle is necessary.

#### 4.2.2 Variability Between Scintillation Cells

As is shown in table 2, the variabilty due to real differences between cells amounts to 2.6% for 1*s_i_*. These differences are attributable to variability in scintillation response and exclude the effect of photomultiplier scintillation counting response, as described in section 2.3.

#### 4.2.3 Statistical Uncertainty Due to Counting

This is normally small due to the large activity transferred and the length of time used to count the sample. For the counting periods and concentrations of Rn-222 used in our laboratory, this uncertainty is usually less than 1%.

#### 4.2.4 Estimate of Volume in Volumetric Method

In this case, the volume was determined in the same manner as for the cell, and the uncertainty was estimated to be 1 cm^3^ in 1300 cm^3^, or 0.08%.

#### 4.2.5 Leakage of Rn-222 From the Vessel Holding the Standard

To minimize this possibility, the vessel was held at negative pressure relative to atmospheric after transfer.

#### 4.2.6 The Pressure of the Air in the Scintillation Cell

Any change in pressure within the scintillation cell will change the range of alpha particles and so alter the number striking the zinc sulphide coating on the cells and hence the cell calibration. Therefore all cells were brought to the same pressure on completion of the transfer of the radon.

#### 4.2.7 Humidity of the Air

This was reduced using a dry ice trap in early work, but did not appear to affect the calibration results and so the practice was discontinued.

#### 4.2.8 Overall Random Uncertainty

In the estimation of overall random uncertainty in the determination of Rn-222 concentration by any one ARL scintillation cell calibrated by the volumetric method, the pertinent components which should be considered were
1)Variability in repeat calibration factor for one cell=2.3% (*s_i_* with *n* = 4) for any cell2)Variability due to cell differences=2.6% (*s_i_* with *n* = 16) for any cell3)Counting statistics during Rn-222 measurement=0.9% (SEOM with *n*=30)where SEOM = standard error of the mean. The overall random uncertainty for the determination of Rn-222 concentration for any cell can be calculated by adding the components as follows:

$${\left(0.023\right)}^{2}+{\left(0.026\right)}^{2}+{\left(0.009\right)}^{2}={\left(0.036\right)}^{2},$$

which is 3.6% and in good agreement with the measured value of 3.4% shown in table 3. The good agreement also indicates that most of the relevant sources of random uncertainty have been considered.

The systematic uncertainty was mainly derived from the total uncertainty of the Ra-226 solution, ignoring the contributions of other minor components. This solution was supplied by Amersham (UK) and traceable to NIST, and from the certificate, the random uncertainty applicable to the source was 0.7% at 3 standard deviations, or 0.2% at one standard deviation, with the systematic uncertainty estimated to be 3.0%. Using the recommendations of Collé et al. [3], the systematic uncertainty in the Rn-222 measurement due to the total uncertainty of the source was calculated as follows:

$${\left(0.002\right)}^{2}+{\left(0.030\right)}^{2}≅{\left(0.030\right)}^{2}$$

which is 3.0%. Hence the total uncertainty for any ARL scintillation cell was calculated from,

$${\left(0.036\right)}^{2}+{\left(0.030\right)}^{2}={\left(0.047\right)}^{2}$$

which is 4.7%, and both the random and systematic uncertainties are contributing comparable amounts to the total uncertainty.

### 4.3 Reduction in Calibration Variability

The results of Rn-222 concentration determined from the new individual calibration factors are shown in table 4; it is clear that the grand *s_i_* has been reduced from 3.4% to 1.8%. Correspondingly, the variability due to differences between cells was reduced from 2.6% to 0.9%, which is comparable with the counting statistics.

## 5. Conclusions

The results of standardization of Rn-222 by ARL using two techniques clearly shows there is no difference between calibrations using the injection or volumetric methods, with the latter providing the reliable transfer of Rn-222. Calibration factors based on the volumetric method resulted in a reduction from 3.4% to 1.8% in the measurement uncertainty for Rn-222 by any ARL scintillation cell.

## Figures and Tables

**Table 1:** Values of calibration factors for one ARL cell using injection and volumetric methods counts × 10^−6^ s^−1^(Bq m^−3^)^−1^

Method	(1)	(2)	(3)	(4)	Mean±1*s_i_*
Injection^a^	2230	2200	2310	2300	2260±52^b^
Volumetric^a^	2240	2340	2240		2273±55^c^

a Difference in Means=0.3%.

b Injection 1*s_i_*    =2.3%.

c Volumetric 1*s_i_*   =2.4%.

**Table 2:** Variability in Rn-222 measurements between ARL scintillation cells exposed to same Rn-222 concentration and analyzed using a calibration factor of unity (Bq m^−3^)

Group	(1)	(2)	(3)	(4)
N1	3290	3350	3430	3470
N2	3480	3570	3450	3470
N3	3350	3500	3480	3500
N4	3410	3520	3690	3470

Grand mean = 3460±90 Bq m^−3^ or 2.6% at 1*s_i_*.

**Table 3:** Rn-222 concentrations calculated using original calibration factors (Bq m^−3^)

Group	(1)	(2)	(3)	(4)
N1	2580	2610	2700	2690
N2	2600	2690	2690	2640
N3	2560	2660	2870	2510
N4	2660	2670	2780	2680

Grand mean=2660±90 Bq m^−3^ or 3.4% for 1*s_i_*.

**Table 4:** Rn-222 concentrations determined using adjusted calibration factors (Bq m^−3^)

Group	(1)	(2)	(3)	(4)
N1	2690	2630	2680	2650
N2	2590	2630	2660	2690
N3	262Q	2600	2730	2690
N4	2540	2680	2670	2690

Grand mean=2650±50 Bq m^−3^ or 1.8% for 1*s_i_*

## Footnotes

1 Certain commercial equipment, instruments, or materials are identified in this paper to specify adequately the experimental procedure. Such identification does not imply recommendation or endorsement by the National Institute of Standards and Technology, nor does it imply that the materials or equipment identified are necessarily the best available for the purpose.
